# Cardiovascular disease and beta-cell function at diagnosis of serologically defined adult-onset type 1 and type 2 diabetes in two Swedish cohorts 15 years apart

**DOI:** 10.1136/bmjopen-2024-095630

**Published:** 2025-07-16

**Authors:** Viveca Ritsinger, Rebecka Gunnarsson, Eva Melin, Magnus Hillman, Anna Stogianni, Sara Holmberg, Karin Johansson, Ann-Sofie Nilsson Neumark, Herbert Krol, Mattias Rööst, Mona Landin-Olsson, Thomas Neumark, Pär Wanby, Maria Thunander

**Affiliations:** 1 Division of Cardiology, Karolinska Institute Department of Medicine Solna, Stockholm, Stockholm County, Sweden; 2 Department of Research and Development, Region Kronoberg, Växjö, Sweden; 3 Medical Faculty; Linköping University, Linköping, Sweden; 4 Institution of Clinical Sciences, Endocrinology and Diabetes, Lund University, Lund, Sweden; 5 Department of Endocrinology, Karolinska University Hospital, Huddinge, Stockholm, Sweden; 6 Department of Medicine and Optometry, Linnaeus University, Kalmar-Växjö, Växjö, Sweden; 7 Primary Care, Region Kalmar, Kalmar, Sweden; 8 Department of Internal Medicine, Kalmar Hospital, Kalmar, Sweden; 9 Department of Clinical Sciences, Lund University, Malmö, Sweden

**Keywords:** Cardiovascular Disease, DIABETES & ENDOCRINOLOGY, Prevalence

## Abstract

**Abstract:**

**Objectives:**

To describe the prevalence of cardiovascular disease (CVD) at the time of diagnosis of adult-onset type 1 (T1D) and type 2 (T2D) diabetes, in a recent cohort and compare to a previous cohort from the same region. Further, to explore factors influencing the prevalence of pre-existing CVD, including age, sex, body mass index (BMI) and C-peptide; in the later cohort also heart failure, hyperlipidaemia, tobacco use and physical activity.

**Design:**

Two prospective cross-sectional cohort studies compared.

**Setting:**

All primary health care centres and hospitals in Kalmar and Kronoberg counties in Southeastern Sweden.

**Participants:**

Adults with newly diagnosed T1D or T2D (classified by combination of islet antibodies and C-peptide) in 1998–2001 and 2016–2017.

**Primary and secondary outcome measures:**

Prevalence of hypertension and CVD at diagnosis of diabetes, and associations with beta-cell function, in two cohorts collected 15 years apart. Further, to explore factors influencing the prevalence of hypertension and CVD, and level of C-peptide.

**Results:**

In patients with newly diagnosed T2D, mean age-at-onset had decreased (66±14.1 years vs 63±12.6, p≤0.001) and mean BMI had increased (29.0±5.4 vs 31.4±5.8 kg/m^2^, p≤0.001). Prevalence of pre-existing myocardial infarction had decreased in both T1D (18% vs 7%, p=0.03) and T2D (25% vs 11%, p≤0.001). Pre-existing hypertension had increased in both T1D (23% vs 40%, p=0.01) and T2D (44% vs 61%, p≤0.001). C-peptide level was lower and was associated with several cardiovascular conditions in newly diagnosed T2D in 2016–2017 (p=0.048 p≤0.001).

**Conclusions:**

Patients with newly diagnosed T2D were younger, with higher BMI, compared with 15 years earlier, a challenge for diabetes care. Prevalence of pre-existing myocardial infarction had decreased notably, in line with, but still less than in the general population; while pre-existing hypertension had increased, in both diabetes types. C-peptide was associated with several cardiovascular conditions in newly diagnosed T2D in the recent cohort, which warrants further investigation.

**STRENGTHS AND LIMITATIONS OF THIS STUDY**

- We were able to include a large sample size of adult patients with newly diagnosed diabetes who were all thoroughly examined in person, in total 207 patients with type 1 diabetes and 2515 patients with type 2 diabetes.
- As the classification of diabetes types was serological, with a combination of islet antibodies and C-peptide level, the number of adults with newly diagnosed clear type 1 diabetes was increased compared with other studies.
- Although most of the methodology was the same in both cohort studies, the collected data differed where the recent cohort included more information regarding lifestyle, metabolic factors and heart failure compared with the previous, limiting a full comparison between the two cohorts.
- As the event rates of some CV conditions were small, there was limited strength for some of the analyses.

## Introduction

 According to Swedish and British studies, two out of three patients with newly diagnosed myocardial infarction (MI) or stroke have either diabetes or a prediabetic state; only 1/3 have normal glucose tolerance.^1^,^4^ Diabetes affects 537 million people worldwide, expected to increase to 643 million by 2030.^5^ In 2016, the total costs of complications of type 2 diabetes (T2D) in Sweden reached €270 million, emphasising the need to investigate the cardiovascular (CV) situation in adults with newly diagnosed, not only established, type 1 diabetes (T1D) and T2D.^6^ T1D is often described with onset during childhood or adolescence but is diagnosed in all adult age groups, from 18 to 100 years, and, as recently more generally recognised, there are more new adult-onset than childhood-onset cases of T1D.^7 8^ Patients with T1D with onset before the age of 30 years in the Swedish National Diabetes Registry (S-NDR) had higher all-cause mortality and twice as high CV mortality as patients without T1D.^9^ Contrary to that, a 15-year follow-up of the population-based Kronoberg incidence study of serologically defined adult-onset diabetes found that standard mortality rates of adult-onset T1D did not differ from the general population in any adult age group.^10^ CV disease (CVD) is the most prevalent complication of T2D, often accompanied by obesity and insulin resistance, where beta-cell function is a derivative of a combination of both constitutional and environmental factors.^11^ Frequently preceding T2D is especially hypertension but also the metabolic syndrome, which further involves visceral obesity, dyslipidaemia and hyperglycaemia.^12^ Individuals with the metabolic syndrome are at high risk of developing both CVD and T2D. Patients with T2D have 2–4 times increased risk of CV events and death compared with the general population.^10 13^ There is a strong association between hyperglycaemia and complications of diabetes such as MI, stroke, peripheral vascular disease and microvascular disease.^14^

Studies of incidence and new onset of T1D usually include children aged 0–15, occasionally 0–19 years. The Kronoberg study DIK (Diabetes Incidence in Kronoberg) 1998–2001 was unique in also including all adults with new diabetes aged 18–100 years, and with the best determination of diabetes type, via blood tests for islet antibodies and C-peptide level.^715^,^17^ The DIK study also includes population-based data on incidence and all new cases of children and adults with T2D, most often examined as a prevalence, and where prospective inclusion of all new adult cases in a geographical area over time is unusual. A new study of newly diagnosed diabetes was conducted in 2016–2017; the Diabetes in Kalmar and Kronoberg (DKK) study.

The prevalence of obesity and T2D has increased in recent decades, imposing increasing burdens on healthcare and new therapies are continuously developed. Early intervention is favourable. Describing the comorbidity and trends in newly diagnosed diabetes is important. The aim of the present study was therefore to describe the prevalence of hypertension and CVD at the diagnosis of diabetes in a recent cohort of adults with newly diagnosed serologically defined T1D or T2D, and associations with beta-cell function; and compare with the prevalence of hypertension and CVD in a similar cohort from Kronoberg 15 years earlier, and explore factors influencing the prevalence of hypertension and CVD, and level of C-peptide.

## Methods

### Study population

This study included data from two prospective cross-sectional cohorts collected 15 years apart. The first cohort, the DIK study 1998–2001 included 1666 patients and 96% (n=1592) had sufficient information regarding previous CV conditions and diabetes type (7). The DKK study 2016–2017 included 1248 patients, 91% (n=1130) had sufficient data for this analysis.^18 19^ Inclusion criteria for both cohorts were patients with newly diagnosed diabetes, aged 18 years and above.^7 18 19^ Exclusion criteria were current gestational diabetes and age below 18 years, and incomplete information regarding pre-existing CV conditions.^7 18 19^ DKK included patients with newly diagnosed diabetes visiting primary healthcare centres (PHCCs, n=55) and all hospitals (n=5) in Kalmar and Kronoberg counties in Southeastern Sweden. Patients were invited, included and examined during ordinary routine or acute visits. The studies were performed according to the Declaration of Helsinki and its amendments. All participants gave informed consent, DKK written; DIK oral.

### Patient and public involvement statement

Patients or the public were not involved in the design, conduct, reporting or dissemination plans of our research.

### Examinations

Variables from the first cohort were: age-at-diagnosis of diabetes (years), sex (M/W), body mass index (BMI) (kg/m^2^), systolic and diastolic blood pressure (standardised procedure, mm Hg) and prevalence of hypertension and other pre-existing CV conditions, as MI/IHD (ischaemic heart disease), stroke/transient ischaemic attack (TIA) and peripheral arterial insufficiency/intermittent claudication (PAI). Clinical data was collected by physicians or trained registered nurses. Intravenous blood samples were collected for central analyses of islet antibodies and C-peptide, for classification of diabetes type. In the second cohort, the same characteristics and information regarding lipids (T-cholesterol, triglycerides, low-density lipoprotein (LDL) and high-density lipoprotein, mmol/L); tobacco use (never, ex tobacco user, snuff only, smoker only, smoker and snuff); physical activity (by the scale used by the S-NDR (never, <1, 1–2, 3–5 times/week, every day); level of haemoglobin A1c (HbA1c) (mmol/mol) at diagnosis of diabetes; and pre-existing heart failure were registered.

### Definition of diabetes

Diabetes was diagnosed according to the WHO/American Diabetes Association (ADA) criteria: fasting glucose, venous or capillary ≥7.0 mmol/L on two different occasions; or p-glucose ≥11.1/≥12.2 mmol/L (venous/capillary) once random or at 120 min during a 75 g oral glucose tolerance test.^17^ All pathological values were confirmed by further testing.

### Classification of diabetes

Patients positive to any islet antibody, or with C-peptide value <0.25 nmol/L were classified as T1D. Patients negative to islet antibodies with C-peptide ≥0.25 nmol/L were classified as T2D. In the DIK study, GADA (glutamic acid decarboxylase antibody) and ICA (islet cell antibody) were analysed; in DKK, only GADA was primarily analysed, but for all patients with C-peptide <0.50 nmol/L also IA–2A (tyrosine phosphatase) and ZnT8 (Zinc transporter 8) antibodies were analysed. Classification of diabetes type using both islet cell antibodies and C-peptide level is described as the best method to discriminate between autoimmune (type 1) and non-autoimmune (type 2) diabetes.^7 15 16 20^

### Laboratory methods

Capillary blood samples were analysed using the HemoCue Glucose System (HemoCue AB, Ängelholm, Sweden) at the PHCCs. Venous blood glucose samples were collected at the hospitals and analysed with routine methods at their respective departments of clinical chemistry. Islet antibodies and C-peptide were centrally analysed at the Diabetes Laboratory, Biomedical Centre, Skane University Hospital, Lund, with commercial kits, according to manufacturers’ instructions. In the first cohort, C-peptide was sampled fasting, in the second random, expecting to evoke a stimulated, potentially greater, response.^21^ Lipids were analysed with a high specificity Olympus AU Clinical Chemistry Analyser (Olympus AU, Tokyo, Japan).

### Statistical analyses

Descriptive statistics, t-test for continuous parameters, and χ² test (Fisher’s exact test and Pearson χ²) for dichotomised variables, for comparisons between groups and comparison of variables between the two cohorts. Non-parametric data was analysed with the Mann-Whitney U-test. All tests were two-sided. p<0.05 was considered statistically significant. When comparing between the cohorts ‘having one or more CVDs’, pre-existing hyperlipidaemia and heart failure were excluded since that information was not collected in the first cohort. Simple logistic regression analyses were performed exploring factors influencing the prevalence of pre-existing hypertension or CVD. Variables with p values ≤0.10 were entered into multiple logistic regressions (Backward Wald) with pre-existing hypertension and the different CVDs as dependent variables. Age at diagnosis, sex, BMI, C-peptide and pre-existing hypertension were independent variables. Tobacco use was controlled for and mentioned where there was an association. The multiple logistic regression models were evaluated by Nagelkerke R square. CIs given are 95%. All analyses were performed with SPSS (Statistical Package for the Social Sciences, Chicago, Illinois, USA) V.26.0.

## Results

Baseline characteristics in patients with newly diagnosed diabetes in the DKK study 2016–2017, in the DIK study 1998–2001 and a comparison between T1D and T2D within each cohort are displayed in table 1.

**Table 1:** Baseline characteristics in patients with newly diagnosed diabetes in Kalmar and Kronoberg 2016–2017 and in Kronoberg 1998–2001, and a comparison between type 1 and type 2 diabetes within each cohort

	Cohort 1998–2001(n=1592)*			Cohort 2016–2017(n=1130)†		
	Type 1 diabetes(n=107)	Type 2 diabetes(n=1485)	P value	Type 1 diabetes(n=100)	Type 2 diabetes(n=1030)	P value
Sex (male)	58 (54)	761 (51)	–	63 (63)	614 (60)	–
Age-at-diagnosis (years)	56 (SD 18.7)	66 (SD 14.1)	<0.001‡	52 (SD 19.0)	63 (SD 12.6)	<0.001‡
BMI (kg/m^2^)§	26.2 (SD 5.2)	29.0 (SD 5.4)	<0.001‡	27.0 (SD 6.2)	31.4 (SD 5.8)	<0.001‡
HbA1c at diagnosis (mmol/mol)¶	–	–	–	77 (SD 32)	59 (SD 23)	<0.001**
C-peptide	0.78 (SD 0.6)	1.48 (SD 0.9)	<0.001**	0.72 (SD 0.6)	1.24 (SD 0.7)	<0.001**
(nmol/L)	0.60 (0.13–2.8)	1.30 (0.26–8.3)		0.49 (0.10–2.7)	1.07 (0.25–5.6)	
GADA			–			–
Negative	20 (19)	1485 (100)		16 (16)	1030 (100)	
Positive	87 (81)	0 (0)		84 (84)	0 (0)	
Systolic blood pressure (mm Hg)††	–	–	–	130 (101–226)	135 (75–215)	0.047‡
LDL (mmol/L)‡‡	–	–	–	3.4 (0.4–6.8)	3.2 (0.9–8.0)	0.91‡
Tobacco§§						0.03¶¶
Never				44 (44)	415 (40)	
Ex tobacco				26 (26)	359 (35)	
Snuff only				18 (18)	102 (10)	
Smoker only				6 (6)	117 (11)	
Smoker and snuff				1 (1)	16 (1.6)	
Physical activity††††						0.06¶¶
Never				6 (6.0)	140 (14)	
<1 time/week				13 (13)	167 (16)	
1–2 times/week				24 (24)	170 (17)	
3–5 times/week				24 (24)	202 (20)	
Every day				24 (24)	288 (28)	
Pre-existing CV condition						
Hypertension***	25 (23)	658 (44)	<0.001†††	40 (40)	628 (61)	<0.001†††
Hyperlipidaemia‡‡‡	–	–	–	18 (18)	380 (37)	<0.001†††
MI/IHD§§§	19 (18)	370 (25)	0.10†††	7 (7.0)	108 (11)	0.30†††
Stroke/TIA¶¶¶	2 (1.9)	112 (7.5)	0.03†††	3 (3.0)	71 (6.9)	0.20†††
PAI	1 (0.9)	39 (2.6)	0.52†††	3 (3.0)	32 (3.1)	1
Heart failure	–	–	–	3 (3.0)	44 (4.3)	0.80†††
One or more CVDs—excludinghypertension.**** And for 2016–2017 excluding HF and hyperlipidaemia.	22 (21)	521 (35)	0.02†††	13 (13)	211 (21)	<0.001†††

Data are n (%), mean (SD) or median (minimum–maximum).

* Number is 1592 except in variable BMI, n=1558.

† Number is 1130 unless otherwise specified.

‡ T-test.

§ N=1082.

¶ N=1095.

** Mann-Whitney U.

†† N=1017.

‡‡ N= 868.

§§ N=1104.

¶¶ Pearson χ².

*** N=1115.

††† Fisher’s exact test.

‡‡‡ N=1020.

§§§ N=1102.

¶¶¶ N=1110.

**** N=1030.

†††† N=1058.

BMI, body mass index; CV, cardiovascular; CVD, cardiovascular disease; GADA, glutamic acid decarboxylase antibody; HbA1c, haemoglobin A1c; HF, heart failure; IHD, ischaemic heart disease; LDL, low-density lipoprotein; MI, myocardial infarction; PAI, peripheral arterial insufficiency; TIA, transient ischaemic attack.

### The DKK cohort 2016–2017 

The DKK cohort 2016–2017 included 1130 patients, 9% (100) with T1D and 91% (1030) with T2D (table 1). Mean age-at-diagnosis was lower for patients with T1D (p≤0.001). Mean BMI and median C-peptide level were higher in patients with T2D (both p≤0.001), as were prevalence of pre-existing hypertension, hyperlipidaemia and having one or more CVDs (all p≤0.001; table 1). There were no significant differences between the 63 men and 37 women with new T1D. Gender proportions were the same in new T2D, 60% (614) men and 40% (416 women). Mean BMI (kg/m^2^) and LDL-cholesterol (mmol/L) were higher among women with T2D than men, 32.0±6.4 vs 30.9±5.3 (p=0.005); 3.5±1.1 vs 3.1±1.1 (p≤0.001) while mean HbA1c (mmol/mol) at diagnosis was higher among men 61.1±24.1 vs 55.5±19.6 (p=0.001). Of men with T2D, 40% were already diagnosed with hyperlipidaemia compared with 33% of the women (p=0.048). Pre-existing MI/IHD (13% vs 6.5%, p≤0.001), PAI (4.6% vs 1.0%, p=0.001) and having one or more CVDs (48% vs 41%, p=0.02) were also more prevalent among men.

### The DIK cohort 1998–2001

**Figure 1:**
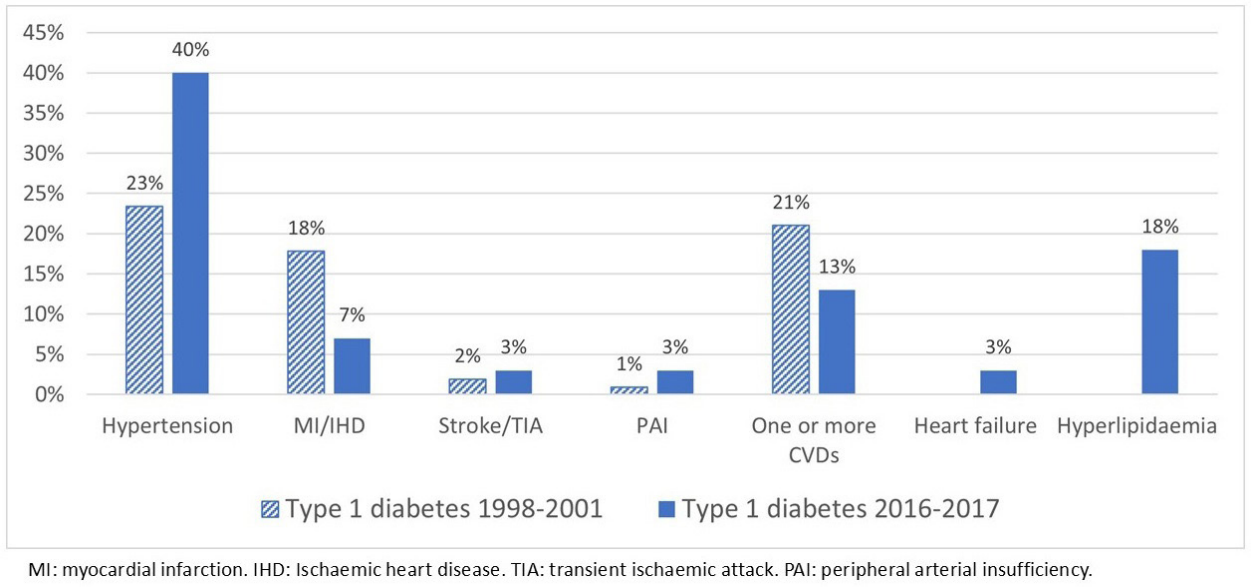
Pre-existing cardiovascular disease (CVD), hypertension and hyperlipidaemia at diagnosis of type 1 diabetes in Kronoberg 1998–2001 and in Kalmar and Kronoberg 2016–2017.

Of the 1592 patients, 7% (107) had T1D, and 93% (1485) had T2D (table 1). Mean age-at-diagnosis, mean BMI and median C-peptide level were higher in patients with newly diagnosed T2D (all p≤0.001). Prevalence of pre-existing hypertension (p≤0.001), stroke/TIA (p=0.03) and already having one or more CVDs (p=0.02) at diagnosis of diabetes was more common in patients with T2D (table 1, figures1^2^). There were no significant differences between men and women with T1D in this cohort either. In women with T2D, mean age-at-diagnosis (67.0±14.4 vs 65.0±13.7 years, p≤0.001), mean BMI (29.4±5.8 vs 28.6±5.0 kg/m^2^, p=0.01) and median C-peptide level (1.30, 0.26–8.10 vs 1.20, 0.26–8.30, nmol/l, p=0.02) were higher than in men. Pre-existing hypertension was more prevalent among women than men (49% vs 40%, p=0.001). The prevalence of pre-existing MI/IHD (28% vs 22%, p=0.008), stroke/TIA (9.1% vs 5.9%, p=0.02) and having one or more CVDs (33% vs 27%, p=0.005) was higher among men than women.

**Figure 2:**
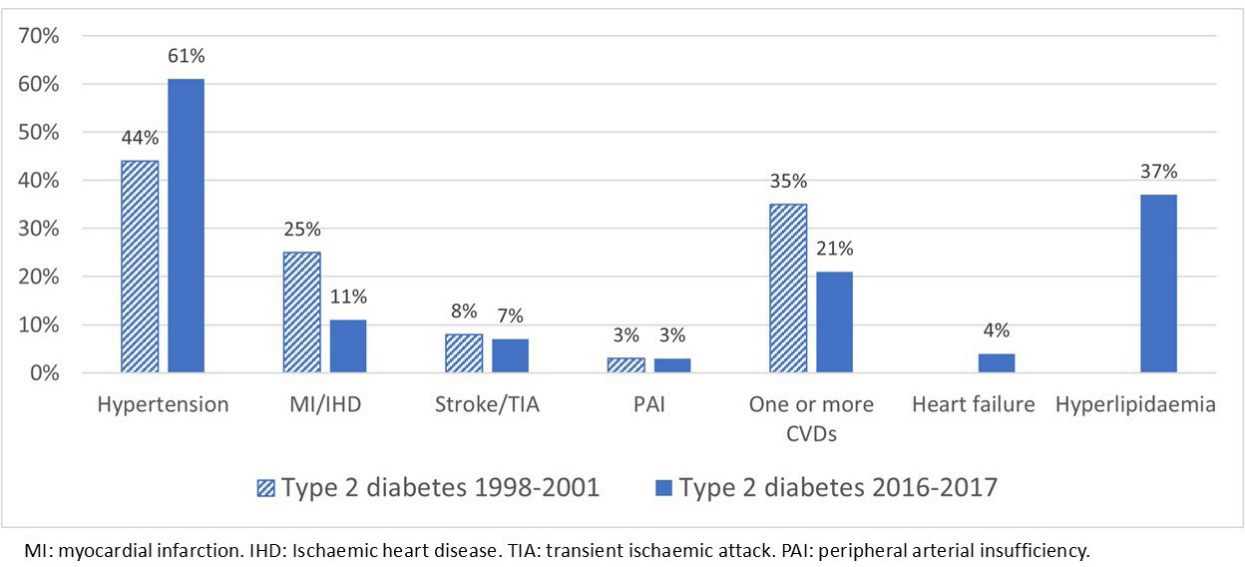
Pre-existing cardiovascular disease (CVD), hypertension and hyperlipidaemia at diagnosis of type 2 diabetes in Kronoberg 1998–2001 and in Kalmar and Kronoberg 2016–2017.

### Comparisons between the DKK cohort 2016–2017 and the DIK cohort 1998–2001

#### Type 1 diabetes

Patients with T1D (100) in the 2016–2017 cohort were compared with the 107 in the 1998–2001 cohort (table 2). There were no differences in age, BMI or C-peptide levels between the cohorts. The prevalence of pre-existing hypertension was higher (p=0.01) and pre-existing MI/IHD was lower (p=0.03) among patients with T1D in the later cohort (table 2, figure 1).

**Table 2:** Comparisons of characteristics in adult patients with newly diagnosed diabetes in Kalmar and Kronoberg 1998–2001 and 2016–2017 by diabetes type

	Type 1 diabetes(n=207)*			Type 2 diabetes(n=2515)†		
	1998–2001(n=107)	2016–2017(n=100)	P value	1998–2001(n=1485)	2016–1017(n=1030)	P value
Age at diagnosis(years)	56 (SD 18.7)	52 (SD 19.0)	0.21‡	66 (SD 14.1)	63 (SD 12.6)66 (17–94)	<0.001‡
BMI (kg/m^2^)§	26.2 (SD 5.2)	27.0 (SD 6.2)	0.29‡	29.0 (SD 5.4)	31.4 (SD 5.8)	<0.001‡
C-peptide (nmol/L)	0.78 (SD 0.6);0.60 (0.13–2.80)	0.72 (SD 0.6);0.49 (0.10–2.71)	0.19¶	1.48 (SD 0.9);1.30 (0.26–8.30)	1.24 (SD 0.7);1.07 (0.25–5.58)	<0.001¶
GADA						
NegativePositive	20 (19)87 (81)	16 (16)84 (84)	0.71**	1485 (100)0 (0.0)	1030 (100)0 (0.0)	–
Pre-existing condition						
Hypertension††	25 (23)	40 (40)	0.01**	658 (44)	628 (61)	<0.001**
MI/IHD‡‡	19 (18)	7 (7.0)	0.03**	370 (25)	108 (11)	<0.001**
PAI/intermittent claudication	1 (0.9)	3 (3.0)	0.36**	39 (2.6)	32 (3.1)	0.54**
Stroke/TIA§§	2 (1.9)	3 (3.0)	0.67**	112 (7.5)	71 (6.9)	0.64**
One or more CVDs¶¶	21 (20)	11 (11)	0.12**	446 (30)	187 (18)	<0.001**

Data are n (%), mean (SD) or median (minimum–maximum).

* Number is 207 unless otherwise specified.

† Number is 2515 unless otherwise specified.

‡ T-test.

§ n=202 (type 1), n=2438 (type 2).

¶ Mann-Whitney U.

** Fisher’s exact test.

†† n=2500 (type 2).

‡‡ n=205 (type 1), n=2489 (type 2).

§§ n=204 (type 1), n=2498 (type 2).

¶¶ n=204 (type 1), n=2488 (type 2).

BMI, body mass index; CVDs, cardiovascular diseases; GADA, glutamic acid decarboxylase antibody; IHD, ischaemic heart disease; MI, myocardial infarction; PAI, peripheral arterial insufficiency; TIA, transient ischaemic attack.

#### Type 2 diabetes

Patients (1030) with newly diagnosed T2D in the 2016–2017 cohort were compared with the 1485 in the 1998–2001 cohort (table 2). During the later period, patients with new T2D were younger, mean 63 vs 66 years (p≤0.001); had higher BMI (kg/m^2^), mean 31.4 vs 29.0 (p≤0.001); but had lower levels of C-peptide (nmol/L), median 1.07 vs 1.30 (p≤0.001). Especially the prevalence of pre-existing MI/IHD was lower, almost 15%, compared with the earlier period (p≤0.001; table 2; figure 2).

#### Factors influencing pre-existing cardiovascular conditions in type 1 diabetes

Age-at-diagnosis AOR (adjusted OR) (1.07 (1.02–1.11), p=0.002) and BMI AOR (1.18 (1.06–1.31), p=0.002) were associated with pre-existing hypertension in patients with new adult-onset T1D in the 2016–2017 cohort when adjusted for C-peptide. These figures were practically the same in the 1998–2001 cohort, age-at-diagnosis AOR 1.08 (1.04–1.11, p≤0.001) and BMI AOR 1.15 (1.02–1.30, p=0.03). Only age-at-diagnosis COR (crude OR) 1.14 (1.04–1.25, p=0.005) was associated with pre-existing MI/IHD in T1D in 2016–2017. This was also true in the previous cohort when adjusted for C-peptide and pre-existing hypertension, AOR 1.06 (1.01–1.12, p=0.01). Neither did any of the factors age-at-diagnosis, sex, BMI, C-peptide or pre-existing hypertension have any influence on the risk of having suffered stroke/TIA before diagnosis of T1D in adults in any of the cohorts. Only age-at-diagnosis was associated with pre-existing PAI COR 1.20 (1.04–1.40, p=0.02) in T1D in 2016–2017. No significant associations were seen in the earlier cohort.

Hyperlipidaemia in newly diagnosed T1D in the recent cohort was associated with hypertension AOR 6.2 (1.46–26.0, p=0.01), adjusted for age-at-diagnosis and C-peptide. No data was available regarding hyperlipidaemia from the first cohort. None of the examined factors had any influence on the prevalence of pre-existing heart failure in the 2016–2017 cohort. No heart failure data was collected in the earlier cohort. Only age-at-diagnosis AOR 1.11 (1.04–1.17, p=0.001) was associated with having one or more pre-existing CVDs in T1D in 2016–2017, adjusting for C-peptide and hypertension. The same magnitude of association was seen in the previous cohort AOR 1.07 (1.02–1.12, p=0.006).

#### Factors influencing pre-existing cardiovascular conditions in type 2 diabetes

Factors influencing pre-existing CV conditions in T2D are depicted in table 3. Age-at-diagnosis AOR 1.06 (p≤0.001), male sex AOR 1.50 (p=0.006), BMI AOR 1.05 (p≤0.001) and C-peptide AOR 1.57 (p≤0.001) were associated with hypertension, in the 2016–2017 cohort. In the previous cohort, age at diagnosis and BMI were associated with hypertension, while C-peptide did not reach statistical significance, and female sex had a positive association. Age-at-diagnosis AOR 1.05 (p≤0.001), male sex AOR 2.2 (p=0.001), C-peptide AOR 1.65 (p≤0.001) and hypertension AOR 1.84 (p=0.02) were associated with MI/IHD in newly diagnosed T2D in 2016–2017. Age at diagnosis, male sex and hypertension were associated with MI/IHD in the previous cohort, while C-peptide was not. In the recent cohort, age at diagnosis AOR 1.04 (p=0.004), C-peptide AOR 1.35 (p=0.048) and pre-existing hypertension AOR 2.9 (p=0.003) were associated with stroke/TIA in new T2D. Age at diagnosis, hypertension and male sex were associated with stroke/TIA in 1998–2001. In the recent cohort, age-at-diagnosis AOR 1.09 (1.04–1.14, p≤0.001), male sex AOR 5.3 (1.79–15.5, p=0.003) and smoking AOR 5.7 (2.3–14.3, p≤0.001) were associated with PAI when adjusted for BMI. In the 1998–2001 cohort, age-at-diagnosis AOR 1.05 (1.02–1.08, p≤0.001) and male sex AOR 2.4 (1.21–4.8, p=0.01) were associated with PAI when adjusting for hypertension. No tobacco habit data was collected in the earlier cohort.

**Table 3:** Factors influencing the prevalence of pre-existing cardiovascular conditions in newly diagnosed type 2 diabetes in two cohorts 15 years apart in Kalmar and Kronoberg

	Cohort 1998–2001				Cohort 2016–2017			
	COR	P value	AOR	P value	COR	P value	AOR	P value
Hypertension								
Age-at-diagnosis	1.03(1.02 to 1.04)	<0.001	1.03(1.03 to 1.04)	<0.001	1.06(1.05 to 1.07)	<0.001	1.06(1.05 to 1.08)	<0.001
Sex (male)	0.71(0.58 to 0.87)	0.001	0.81(0.65 to 1.00)	0.05	1.24(0.96 to 1.61)	0.10	1.50(1.20 to 1.99)	0.006
BMI	1.03(1.01 to 1.05)	0.003	1.05(1.03 to 1.08)	<0.001	1.02(1.00 to 1.04)	0.08	1.05(1.02 to 1.08)	<0.001
C-peptide	1.27(1.13 to 1.43)	<0.001	1.12(0.98 to 1.26)	0.11	1.85(1.50 to 2.30)	<0.001	1.57(1.24 to 1.98)	<0.001
MI/IHD								
Age-at- diagnosis	1.07(1.05 to 1.08)	<0.001	1.07(1.05 to 1.08)	<0.001	1.06(1.04 to 1.08)	<0.001	1.05(1.03 to 1.08)	<0.001
Sex (male)	1.38(1.09 to 1.75)	0.007	1.78(1.37 to 2.30)	<0.001	2.20(1.41 to 3.50)	0.001	2.20(1.35 to 3.50)	0.001
BMI	0.95(0.92 to 0.97)	<0.001	0.98(0.96 to 1.01)	0.25	0.98(0.95 to 1.02)	0.38	–	–
C-peptide	1.82(1.05 to 1.34)	0.006	1.01(0.88 to 1.15)	0.94	1.87(1.47 to 2.40)	<0.001	1.65(1.28 to 2.10)	<0.001
Hypertension	1.82(1.43 to 2.30)	<0.001	1.57(1.21 to 2.00)	0.001	3.00(1.78 to 4.90)	<0.001	1.84(1.09 to 3.10)	0.02
Stroke/TIA								
Age at diagnosis	1.08(1.06 to 1.10)	<0.001	1.08(1.06 to 1.10)	<0.001	1.05(1.02 to 1.07)	<0.001	1.04(1.01 to 1.06)	0.004
Sex (male)	1.58(1.06 to 2.40)	0.02	2.30(1.48 to 3.50)	<0.001	1.37(0.83 to 2.30)	0.22	–	–
BMI	0.93(0.89 to 0.97)	<0.001	0.97(0.93 to 1.03)	0.20	1.03(0.99 to 1.07)	0.15	–	–
C-peptide	1.11(0.92 to 1.35)	0.27	–	–	1.59(1.20 to 2.11)	0.001	1.35(1.00 to 1.82)	0.048
Hypertension	2.30(1.56 to 3.50)	<0.001	2.10(1.40 to 3.30)	<0.001	4.00(2.00 to 8.00)	<0.001	2.90(1.44 to 5.80)	0.003

CIs are 95%. Nagelkerke R square = 0.081/0.186, 0.186/0.147, 0.165/0.086.

AOR, adjusted OR; BMI, body mass index; COR, crude OR; IHD, ischaemic heart disease; MI, myocardial infarction; TIA, transient ischaemic attack.

Hyperlipidaemia was associated with hypertension AOR 4.0 (2.9–5.5, p≤0.001) and C-peptide AOR 1.28 (1.05–1.57, p=0.02) in the 2016–2017 cohort, adjusted for age-at-diagnosis and sex. No data was available regarding hyperlipidaemia in the first cohort.

Pre-existing heart failure was recorded in the recent cohort and was associated with age-at-diagnosis AOR 1.11 (1.07–1.15, p≤0.001) and male sex AOR 2.1 (1.01–4.2, p=0.047) in T2D, adjusted for hypertension and C-peptide. No data on heart failure was collected in the earlier cohort.

Age-at-diagnosis AOR 1.02 (1.01–1.04, p≤0.001), male sex AOR 1.37 (1.03–1.82, p=0.03), C-peptide AOR 1.44 (1.17–1.77, p=0.001) and hypertension AOR 3.5 (2.6–4.7, p≤0.001) were associated with having one or more pre-existing CVDs in new T2D in the recent cohort. Similar results were seen previously, age-at-diagnosis AOR 1.08 (1.06–1.09, p≤0.001), male sex AOR 1.91 (1.48–2.5, p≤0.001) and hypertension AOR 1.69 (1.31–2.2, p≤0.001) associated with one or more pre-existing CVDs, except for C-peptide, which was associated crudely, but not when adjusted.

## Discussion

In this large observational study of two cohorts with newly diagnosed diabetes from the same geographical area during different time periods, there were three major findings. First, patients with newly diagnosed T2D were younger and had higher BMI compared with 15 years earlier. Second, the prevalence of MI/IHD already at the time of diagnosis of diabetes had decreased substantially during the 15-year period both among patients with T1D and T2D, although not to the same extent as in the general population. Third, the level of C-peptide was associated with the prevalence of several pre-existing CV conditions among patients with newly diagnosed T2D in the recent cohort.

In the recent cohort of adult patients with newly diagnosed T1D or T2D, pre-existing hypertension and hyperlipidaemia were more prevalent in T2D. Further, having one or more CV conditions at diagnosis of diabetes was more prevalent in T2D compared with T1D, in accordance with the known higher risk of CV risk factors and conditions in T2D.^13 22^ Patients with adult-onset T1D were younger than those with newly diagnosed T2D, in line with the findings of our previous cohort, and with some of the few other incidence studies of T1D in adults.^23^ We found, in agreement with our previous population-based study, that T1D was diagnosed in all adult age groups, not only among children and adolescents.^7 8 16^

BMI and systolic blood pressure were higher in patients with T2D. This may be explained by BMI, indirect visceral obesity and hypertension being part of the metabolic syndrome, an established major risk factor for T2D.^12 24^ Many adults with new T1D were also overweight, and most with T2D were obese, in accordance with the 2019 annual report from the S-NDR.^25^ The proportion of obese patients with diabetes is increasing, in line with what we and others have previously demonstrated.^15 25 26^

The distributions of age and BMI within the different cohorts were thus similar when the diabetes types were compared. Patients with T1D were younger at the time of diagnosis, and patients with T2D had a higher BMI—but the values changed over time. Age at onset decreased, and BMI increased in both types. This inverse relationship, that adults with early onset of T2D were more obese, was in accordance with some previous observations.^27^ The lower beta-cell function in that group has previously been demonstrated in experimental studies.^28 29^

The prevalence of pre-existing MI/IHD at diagnosis of diabetes had decreased by 11% among patients with new T1D and almost 15% in new T2D. There was a greater reduction, 38%, during the similar period, for the Swedish population in general than in this population including only patients with diabetes.^30^ They do, however, follow the same descending trend, with clearly decreasing incidence of acute MI.

Simultaneously with the decrease in MIs, the prevalence of hypertension at diagnosis had increased substantially in both diabetes types. Also means BMI had increased in T2D, possibly related to the fact that hypertension often is associated with higher BMI, and possibly a higher early detection rate of hypertension.^24^ Despite the increasing prevalence of hypertension and higher BMI, a worse risk profile, fewer had already suffered MI/IHD at the time of diagnosis, likely explained by advances in the healthcare. Enhanced treatment of risk factors as antihypertensives, lipid-lowering mainly with statins, use of novel cardioprotective glucose-lowering drugs with multiple effects on blood pressure, lipids and CVD; better glucose control and lifestyle interventions are likely responsible for reducing the CV risk.^1431^,^34^ Another contributing factor decreasing MI/IHD at diagnosis could be the smoking cessation trend in Sweden. The proportion of daily smokers almost halved from 20% to 11% in 2000–2016 (16–84 years) in the Swedish population.^35^

Prevalence of one or more CVDs at diagnosis of diabetes had decreased numerically over time in both diabetes types, but significantly only in T2D, possibly due to the limited number of adults with new T1D, to be expected from the usual relation of incidence between T1D and T2D.^7^ The decrease was in accordance with Swedish nationwide health registry data 1998–2014 showing reductions in the incidence of CV complications among adults with established T1D and T2D.^13^ The reduction of pre-existing MI/IHD in this study was also in line with those registry data, whereas stroke/TIA and PAI had not decreased, more difficult to assess due to the low number of those events in this population.

Our findings of higher age-at-diagnosis, male sex and hypertension being associated with several pre-existing CV conditions in both cohorts were in line with established risk factors for CVD.^36^ BMI, however, was not at all associated with the prevalence of pre-existing CVD but with hypertension. Various studies have reported obesity to be an independent risk factor for CVD.^24 37^ Recently, an obesity paradox has been described, suggesting that patients with higher BMI have lower or similar CV mortality.^38^ Also, other measures than BMI have been suggested, with waist-to-hip measurement, or waist-to-height, as better alternatives to estimate obesity and the CV risk.^39 40^ C-peptide was associated with hypertension and several CVDs in T2D in the recent but not the earlier cohort. Median C-peptide levels had decreased in adult patients with newly diagnosed T2D from 1998 to 2001 to 2016 to 2017. That lower beta-cell function is associated with higher BMI and worse CV health has been experimentally shown before.^28 29^ Possible other reasons may be CV risk factors such as age-at-diagnosis, BMI, metabolic and lifestyle factors in the recent cohort. Age at onset of new T2D was lower in the recent cohort, and BMI was higher. C-peptide levels usually increase with age and with BMI.^15 29^ In the recent cohort, C-peptide was sampled randomly, expected to result in higher levels, compared with fasting C-peptide in the earlier cohort.^15 21^ Despite higher BMI, randomly sampled C-peptide levels were lower in the recent cohort. All this implies that the recent cohort contained a group of patients in worse condition, with greater beta-cell dysfunction, in line with experimental findings.^28^

Our results also showed that the prevalence of MI/IHD had decreased during the 15-year period. How C-peptide levels in patients with new T2D may contribute to CVD is not clear. Epidemiological studies have shown that C-peptide levels were associated with CVD and CV mortality, both among persons without diabetes and with T2D.^41^,^44^ We found a possible association between C-peptide levels and pre-existing CVD. The situation is complex, however, as multiple factors contribute to the CV risk. Other metabolic or lifestyle factors which were not examined in this study might be involved. Further studies are warranted regarding pre-existing CV conditions among patients with newly diagnosed diabetes and their associations with level of C-peptide and other metabolic conditions.

### Strengths and limitations

The major strength of this study was the large sample size of adult patients with new T2D, including in total 2515 patients; and of the less prevalent adult-onset T1D, 207 patients, that were all thoroughly examined in person. Second, the classification of diabetes types was serological, with a combination of islet antibodies and C-peptide, considered the best way to discriminate between T1D and T2D.^7 15 16 20^ This increases the number of adults with newly diagnosed clear T1D compared with other studies. Third, most of the methodology was the same in both cohort studies, enabling comparisons. Limitations of the study are that the number of adults with newly diagnosed T1D was lower, which reflects the incidence rates of T1D and T2D.^7^ The collected data also differed, since the recent cohort included more information regarding lifestyle, metabolic factors and heart failure compared with the previous. Event rates of some CV conditions were small, which resulted in limited strength for some of the analyses. Still, despite this, a number of significant associations could be established.

## Conclusions

In conclusion, we found that the patients with newly diagnosed T2D were younger and had higher BMI compared with 15 years earlier. A small increase in BMI, within the overweight range, was also seen in adults with newly diagnosed T1D; both constitute a challenge for diabetes care. Notable was that the prevalence of pre-existing MI/IHD had decreased substantially during the 15-year period, in line with, but to a lesser magnitude, than in the general Swedish population; while the prevalence of pre-existing hypertension had increased, both among patients with newly diagnosed T1D and T2D. We also found that the level of C-peptide in patients with new T2D was lower in the more recent cohort and was associated with the prevalence of several pre-existing CV conditions, which warrants further investigation.

## Data Availability

The data sets are not publicly available as the ethical permission did not include that. However, data can be made available from the corresponding author upon reasonable request.
