# Supplementary material for: Cardiovascular disease and beta-cell function at diagnosis of serologically defined adult-onset type 1 and type 2 diabetes in two Swedish cohorts 15 years apart
Source: BMJ Open. 2025 Jul 16;15(7):e095630. doi: 10.1136/bmjopen-2024-095630 (PMC12273147; doi:10.1136/bmjopen-2024-095630)
Supplement: Supplementary file 1 [file bmjopen-2024-095630.reviewer_comments.pdf]

PEER REVIEW HISTORY

BMJ Open publishes all reviews undertaken for accepted manuscripts. Reviewers are asked to complete a checklist review form and are provided with free text boxes to elaborate on their assessment. These free text comments are reproduced below.

ARTICLE DETAILS

Title (Provisional)

Cardiovascular disease and beta-cell function at diagnosis of serologically defined adult-onset type 1 and type 2 diabetes in two Swedish cohorts 15 years apart

Authors

Ritsinger, Viveca; Gunnarsson, Rebecka; Melin, Eva; Hillman, Magnus; Stogianni, Anna; Holmberg, Sara; Johansson, Karin; Nilsson Neumark, Ann-Sofie; Krol, Herbert; Rööst, Mattias; Landin-Olsson, Mona; Neumark, Thomas; Wanby, Pär; Thunander, Maria

---

VERSION 1 - REVIEW

Reviewer 1

Name Hollander, Monika

Affiliation University Medical Center Utrecht, Julius Center for Health Sciences and Primary Care

Date 13-Apr-2025

COI None

Dear Authors,

Thank you for this well written and comprehensive study on the differences in characteristics of newly diagnosed diabetes patients in two cohorts 15 years apart.

I think that the paper is of interest to the readers of the BJM open, as it elaborates on trends in diabetes.

In order to increase the impact of the article I have some suggestions and minor remarks:

1. The introduction and discussion could gain strength if the authors elaborate on why they want to assess trends in characteristics of newly diagnosed DM patients - so what is the clinical relevance for health care workers or prevention activities? If no direct translation into practice is yet possible, the authors could explain why.

2. I was puzzled by table 1 and 2, where the same data is shown twice, but with different comparisons. I suggest to present the data only once, and design a table in which the P values for both comparisons are depicted.

3. Minor detail: some figures between the brackets have different number of decimals, could be more uniform.

Reviewer **2**

Name **Rana, Rishabh**

Affiliation **Shaheed Nirmal Mahto Medical College and Hospital, Community Medicine**

Date **05-May-2025**

COI **None**

Thanks for letting us review this manuscript which is an exhaustive one. On going through the whole document I am pointing out one thing . In the figures as well as table it can be seen that HTN holds a special place. In you introduction part i would recommend special mention of either co-occurence of HTN and Diabetes or exploring their association as it has been noted globally. In discussion part it should be dealt separately . Some of the studies at country level are given for your perusal . 1. Rana RK, Jha RR, Sinha R, Kumar D,

Jaiswal R, Patel U, et al. (2024) Correlates of

diabetes mellitus and hypertension in India:

Change as evidenced from NFHS- 4 and 5 during

2015–2021. PLoS ONE 19(7): e0305223. https://

doi.org/10.1371/journal.pone.0305223. 2.Muhammad Haroon Stanikzai, Mohammad Essa Amirzada, Niamatullah Ishaq, Kamil Ahmad Kamil, Zabihullah Anwary, Asadullah Farzad, Ahmad Haroon Baray & Hadia Sayam. (2025) Prevalence of Hypertension and Its Associated Factors Among Patients with Type 2 Diabetes in Southern Afghanistan: A Multi-Center Cross-Sectional Study. Diabetes, Metabolic Syndrome and Obesity 18, pages 715-725.3.Geldsetzer, P., Manne-Goehler, J., Theilmann, M., Davies, J. I., Awasthi, A., Vollmer, S., Jaacks, L. M., Bärnighausen, T., & Atun, R. (2018). Diabetes and Hypertension in India: A Nationally Representative Study of 1.3 Million Adults. JAMA internal medicine, 178(3), 363–372. https://doi.org/10.1001/jamainternmed.2017.8094

VERSION 1 - AUTHOR RESPONSE

Reviewer: 1
Dr. Monika Hollander, University Medical Center Utrecht Comments to the Author:
Dear Authors,

Thank you for this well written and comprehensive study on the differences in characteristics of newly diagnosed diabetes patients in two cohorts 15 years apart. I think that the paper is of interest to the readers of the BJM open, as it elaborates on trends in diabetes.

Reply: We are grateful for this acknowledgement and recognition of our work.

In order to increase the impact of the article I have some suggestions and minor remarks:

Comment 1: The introduction and discussion could gain strength if the authors elaborate on why they want to assess trends in characteristics of newly diagnosed DM patients - so what is the clinical relevance for health care workers or prevention activities? If no direct translation into practice is yet possible, the authors could explain why.

Reply: We appreciate this suggestion and have elaborated on the clinical relevance in the introduction in the revised manuscript. New text reads (page 6): "*The prevalence of obesity and type 2 diabetes have increased the recent decades, imposing increasing burdens on health care, and new therapies are continuously developed. Early intervention is favourable why describing the comorbidity and trends in newly diagnosed diabetes is important.*"

Comment 2: I was puzzled by table 1 and 2, where the same data is shown twice, but with different comparisons. I suggest to present the data only once and design a table in which the P values for both comparisons are depicted.

Reply: We appreciate this suggestion and the ambition to improve our manuscript. However, we believe as Table 1 describes the comparison between Type 1 diabetes and Type 2 diabetes characteristics *within* each time period, and Table 2 compares the characteristics for each diabetes type in *different* time periods, we have left the tables unchanged to avoid ambiguity.

Comment 3: Minor detail: some figures between the brackets have different number of decimals, could be more uniform.

Reply: We appreciate this comment and have therefore updated the tables where we have strived to be consistent with the decimals. Percent is written with one decimal if <10 and none if >10. Standard deviation is given with one decimal. Regarding C-peptide the number of decimals relates to clinical relevance. All confidence intervals are given with two decimals.

Reviewer: 2
Dr. Rishabh Rana, Shaheed Nirmal Mahto Medical College and Hospital Comments to the Author:

Comment 1: Thanks for letting us review this manuscript which is an exhaustive one. On going through the whole document I am pointing out one thing. In the figures as well as

table it can be seen that HTN holds a special place. In you introduction part i would recommend special mention of either co-occurence of HTN and Diabetes or exploring their association as it has been noted globally. In discussion part it should be dealt separately . Some of the studies at country level are given for your perusal .

1. Rana RK, Jha RR, Sinha R, Kumar D, Jaiswal R, Patel U, et al. (2024) Correlates of diabetes mellitus and hypertension in India: Change as evidenced from NFHS- 4 and 5 during 2015–2021. PLoS ONE 19(7): e0305223. https://doi.org/10.1371/journal.pone.0305223.

2.Muhammad Haroon Stanikzai, Mohammad Essa Amirzada, Niamatullah Ishaq, Kamil Ahmad Kamil, Zabihullah Anwary, Asadullah Farzad, Ahmad Haroon Baray & Hadia Sayam. (2025) Prevalence of Hypertension and Its Associated Factors Among Patients with Type 2 Diabetes in Southern Afghanistan: A Multi-Center Cross-Sectional Study. Diabetes, Metabolic Syndrome and Obesity 18, pages 715-725.

3.Geldsetzer, P., Manne-Goehler, J., Theilmann, M., Davies, J. I., Awasthi, A., Vollmer, S., Jaacks, L. M., Bärnighausen, T., & Atun, R. (2018). Diabetes and Hypertension in India: A Nationally Representative Study of 1.3 Million Adults. JAMA internal medicine, 178(3), 363–372. https://doi.org/10.1001/jamainternmed.2017.8094

Reply: We are very grateful for this suggestion and have enhanced the expression of hypertension and the association of hypertension with diabetes in the Introduction as well as in the Discussion. We have also considered the proposed articles but as they reflect how prevalence of hypertension varies in different diabetes subgroups, they are deemed less applicable here as we have not performed such subgroup analyses.
